# A new southern limit for the distribution of African great apes: sympatric western lowland gorilla (*Gorilla gorilla gorilla*) and central chimpanzee (*Pan troglodytes troglodytes*) confirmed in Mayombe National Park, Angola

**DOI:** 10.1007/s10329-026-01252-5

**Published:** 2026-04-27

**Authors:** Mariana Cravo-Mota, Eduardo Kivete Lutondo, João Mavinga Mbenza, Graça Dala Catuti, Tatiana Simão, Dionísia Segunda, Pedro Vaz Pinto, Nuno Ferrand, Paulo Gama Mota, Susana Carvalho

**Affiliations:** 1 CIBIO Centro de Investigação Em Biodiversidade E Recursos Genéticos, InBIO Laboratório Associado, Universidade do Porto, Campus de Vairão, Vila do Conde, Portugal; 2 BIOPOLIS Program in Genomics, Biodiversity and Land Planning, CIBIO, Campus de Vairão, Vairão, 4485 - 661 Portugal; 3 Fundação Kissama, Luanda, Angola; 4 Maiombe National Park, Cabinda, Angola; 5 INBAC Instituto Nacional da Biodiversidade e Áreas de Conservação, Luanda, Portugal; 6 Universidade de Coimbra, Coimbra, Portugal; 7 Gorongosa National Park, Sofala, Mozambique; 8 Oxford University, Oxford, UK; 9 Faculdade de Ciências, Universidade do Porto, Porto, Portugal

**Keywords:** African Great Apes, Gorillas, Chimpanzees, Sympatric species, Mayombe, Cabinda

## Abstract

The distribution of African great apes has remained unconfirmed regarding their southern limit, particularly on the western side of the continent. IUCN maps include the Mayombe forest of Angola as part of the estimated distribution of western lowland gorillas (*Gorilla gorilla gorilla*) and central chimpanzees (*Pan troglodytes troglodytes*). However, until now no published evidence-based records had confirmed the continued presence of both species. The Mayombe forest is a key biodiversity hotspot and a potentially important stronghold for the conservation of great ape populations in Africa. Here, we report the first systematic evidence of both species in the Mayombe National Park, Cabinda, Angola. In 2023, a grid of camera traps was systematically deployed, producing the first visual records of gorillas and chimpanzees. Building on these findings, in 2024, a pilot survey including *ad libitum* field observations was carried out along exploratory trails to maximise data collection. The combination of these records identified a hotspot of great ape activity where six transects were established, and systematic direct and indirect evidence was documented. Chimpanzees were recorded more times across a broader range of evidence categories, while gorillas appeared less and seemed more spatially restricted. Notably, both species were detected at overlapping sites but never simultaneously, indicating sympatric coexistence with spatio-temporal partitioning. These findings confirm the southernmost predicted distribution of both species for this part of Africa, filling critical gaps in the understanding of great ape evolution and biogeography, and providing a baseline for the first demographic and ecological census of great apes in Angola.

## Introduction

The great apes of Central Africa, the western lowland gorilla (*Gorilla gorilla gorilla*, Critically Endangered) and the central chimpanzee (*Pan troglodytes troglodytes*, Endangered) (IUCN 2024), are two of the most emblematic species of the Congo Basin, one of Africa’s most biodiverse and ecologically rich regions (Beja et al. 2019). Although their populations are undergoing severe declines due to habitat loss, hunting, and disease (Junker et al. 2012; Strindberg et al. 2018), the Congo Basin still holds the largest remaining great ape populations and continues to represent a global priority for tropical biodiversity conservation (Strindberg et al. 2018; IUCN 2014; Fünfstück and Vigilant 2015). Over recent decades, research across Central Africa has expanded our understanding of ape ecology, behaviour, and conservation (Morgan and Sanz 2006; Head et al. 2011; Yamagiwa and Basabose 2014; Strindberg et al. 2018; Plumptre et al. 2021). However, several key areas, where access has been historically limited by political instability, logistical challenges, accessibility, or lack of research infrastructure remain poorly studied (Fünfstück and Vigilant 2015; Mfossa et al. 2022).

**Fig. 1:**
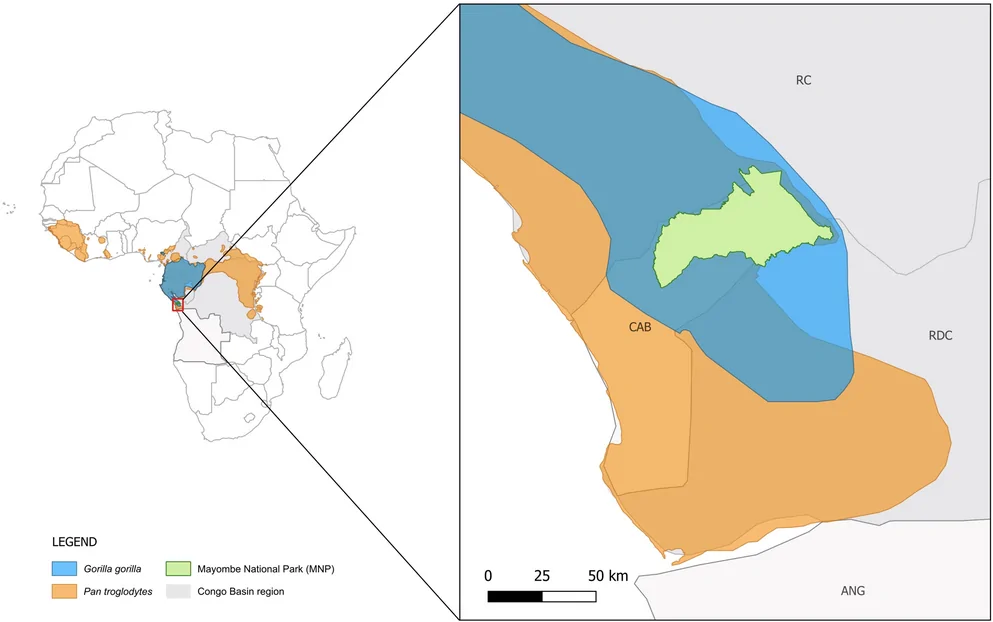
Map of Mayombe National Park (MNP), Cabinda, Angola within the transboundary Mayombe forest shared with Gabon, Republic of Congo, and the Democratic Republic of Congo, western Congo Basin. Shaded areas represent the currently recognized distribution of *Pan troglodytes* and *Gorilla gorilla* based on IUCN Red List spatial data. This map is intentionally generalized to avoid disclosure of sensitive locations

The Mayombe forest, a vast transboundary rainforest extending approximately 10,000 km² across Gabon, Republic of Congo, Democratic Republic of Congo, and into Cabinda (Angola), forms the westernmost extension of the Guineo-Congolian biome. Its dense, humid forests connect the Congo Basin to the Atlantic coast, creating an important ecological corridor that supports high biodiversity and secures genetic exchange across international borders. In the southwestern portion of this region, with 2000 km², lies the Mayombe National Park (MNP), one of the last intact forest blocks in Angola (Fig. 1). Despite its proximity to well documented great ape populations in Gabon and the Republic of Congo (Morgan and Sanz 2006; Devos et al. 2008; Strindberg et al. 2018; Mfossa et al. 2022), the continued presence of gorillas and chimpanzees in the MNP has remained unconfirmed. Historical records, NGO reports and IUCN distribution maps have all pointed towards their occurrence, but no empirical evidence-based study had verified it until now (Ferreira et al. 1945; Ron 2005, 2010, 2020; IUCN 2014). As a result, the MNP represents one of the least studied *areas* for African great apes and a critical gap in our understanding of their current distribution and conservation status.

The absence of confirmed records from MNP is particularly significant given that this region lies within the zone of potential overlap between the western lowland gorilla and the central chimpanzee. Across Central Africa, both species occupy broadly overlapping ranges, although confirmed sites of sympatric coexistence remain relatively rare (Tutin et al. 2005; Strindberg et al. 2018; Mfossa et al. 2022; Sanz et al. 2022). Where both species occur together, coexistence is primarily mediated by spatial and temporal partitioning driven by differences in diet, ranging behaviour, and habitat use (Morgan and Sanz 2006; Head et al. 2011; Oelze et al. 2014). The nature of interspecific interactions varies across sites, ranging from temporal avoidance to occasional tolerance or antagonism, depending on local ecological conditions and resource availability (Deschner and Pika 2021; Sanz et al. 2022). Each new locality where sympatry is confirmed provides important insight into the ecological and evolutionary context of great ape coexistence.

In this context, confirming the presence and understanding how both gorillas and chimpanzees coexist at the MNP provides not only a rare opportunity to investigate patterns of sympatry, ecological adaptation, and behavioural flexibility at the southwestern edge of the Guineo–Congolian forest, but also firmly places Angola on the map of African great ape research. This underscores the urgent need to establish a permanent research station in MNP as a focal point for long-term monitoring and conservation initiatives, thereby integrating Angola into the regional conservation network for the great apes. This paper reports the first systematic documentation of great apes in Angola, based on surveys conducted in 2023 and 2024.

## Methods

### Study area

The study was carried out in the MNP, situated in the Cabinda Province of Angola (approximately 4°46′12″S, 12°35′6″E). The Park covers around 2000 km² and represents the southernmost extension of the Guineo-Congolian rainforest biome, forming part of the transboundary Mayombe forest. This ecosystem is characterized by dense evergreen forest with closed-canopy vegetation, swamp and secondary forest patches along river valleys, and a highly sinuous topography with steep slopes. Elevation ranges from 200 to 800 m and annual rainfall typically between 1500 and 2200 mm, resulting in a perennially humid environment that poses considerable logistical challenges for fieldwork (Ron 2005; Beja et al. 2019).

The vegetation composition includes species from the families Fabaceae, Sapotaceae, Urticaceae, and Zingiberaceae, typical of lowland Guineo–Congolian forests (Linder et al. 2012; Sosef et al. 2017; Beja et al. 2019; Banganga et al. 2020). Human presence within the park remains limited, although selective logging and small-scale agricultural activity occur along its periphery (Ron 2005; Buza 2010).

Our study focused on a 60 km² area located in the central-western sector of MNP. This site was selected due to its relatively low human disturbance and high habitat heterogeneity, making it suitable for the deployment of camera traps and subsequent surveys.

### Survey design and data collection

Fieldwork was conducted between January 2023 and October 2024, following IUCN best practice guidelines for great ape monitoring (Blom et al. 2000; Kühl et al. 2008). Data collection employed a two-phase approach combining systematic camera trapping with targeted field surveys to document the presence and distribution of great apes in the MNP.

In the first phase (January-December 2023), 20 camera traps were deployed across the study area, of which 17 remained operational throughout the survey period. Cameras were positioned systematically at approximately 2 km intervals, with minor adjustments due to terrain heterogeneity, and operated continuously for 12 months (Meek et al. 2014; Kühl et al. 2008).

Although the grid was originally established to assess the occurrence and distribution of the African golden cat (*Caracal aurata*), it provided the first independent visual confirmations of western lowland gorillas and central chimpanzees within the MNP.

Building on these findings, a pilot survey was conducted between June and July 2024, combining *ad libitum* observations and reconnaissance walks covering approximately 140 km to identify additional signs of ape presence within the broader camera trap grid. Surveys were conducted along exploratory trails, including both pre-existing paths and minimally cleared forest routes, to maximize detection opportunities. The integration of *ad libitum* observations and camera trap detections enabled the identification of a great ape activity hotspot within the study area.

Within this hotspot, six line transects were established between August and October 2024, five oriented north–south and one east–west, each measuring 1.5 km in length. Along these transects, both direct (visual encounters, vocalizations) and indirect (nests, feeding traces, faeces, footprints, and trails) evidence of chimpanzees and gorillas were systematically recorded following standard great ape survey protocols (Kühl et al. 2008; Plumptre et al. 2021). For nest records, perpendicular distance to the transect line, nest height, and tree species were recorded whenever possible; these data were collected to support future demographic and ecological analyses. Species identification was based on diagnostic characteristics such as nest architecture, faecal morphology, and species-specific feeding signs (Morgan and Sanz 2006; Head et al. 2011). All observations were independently cross-checked by two experienced observers and validated through consensus, when cross-validation was not possible, records were classified as *“great ape*”. Additional habitat data, including vegetation type, phenology, and fruit availability, were also recorded along each transect.

All research activities were conducted under official permits issued by the Instituto Nacional da Biodiversidade e Áreas de Conservação (INBAC), with logistical and field support provided by the Mayombe National Park Administration and Universidade 11 de Novembro.

### Data analysis

All camera trap data were organized and processed using *R* (v4.3.2) and *Microsoft Excel 365* for metadata extraction, including species identification, date, time, and GPS coordinates. Each record of chimpanzee and gorilla detection was georeferenced and verified to prevent duplication of events, following standardized protocols to ensure data independence (O’Brien et al. 2010; Kühl et al. 2008; Debetencourt et al. 2023). An *independent event* was defined as a sequence of photographs of the same individual or group separated by at least 30 min (Burton et al. 2015; Rovero and Zimmermann 2016). Only independent events were retained for analysis.

Spatial data were mapped in *QGIS 3.44* (QGIS Development Team 2024). Detection points for both species were plotted to visualize their spatial distribution across the camera grid. Sites where both chimpanzees and gorillas were detected were classified as potential overlap zones, representing areas of sympatric occurrence.

For the transect data, all direct and indirect observations were georeferenced using handheld GPS units (*Garmin eTrex 30x*) and compiled into a spatial database. Records were grouped by evidence type (nest, feeding trace, faeces, vocalization, footprint, and visual encounter) and by species. Descriptive statistics were generated to summarize encounter frequencies per transect and evidence type.

Given the limited sample size and temporal scope of the study, analyses focused exclusively on confirming species presence, mapping spatial distribution, and identifying relative patterns of overlap between chimpanzees and gorillas within the study area.

## Results

**Fig. 2:**
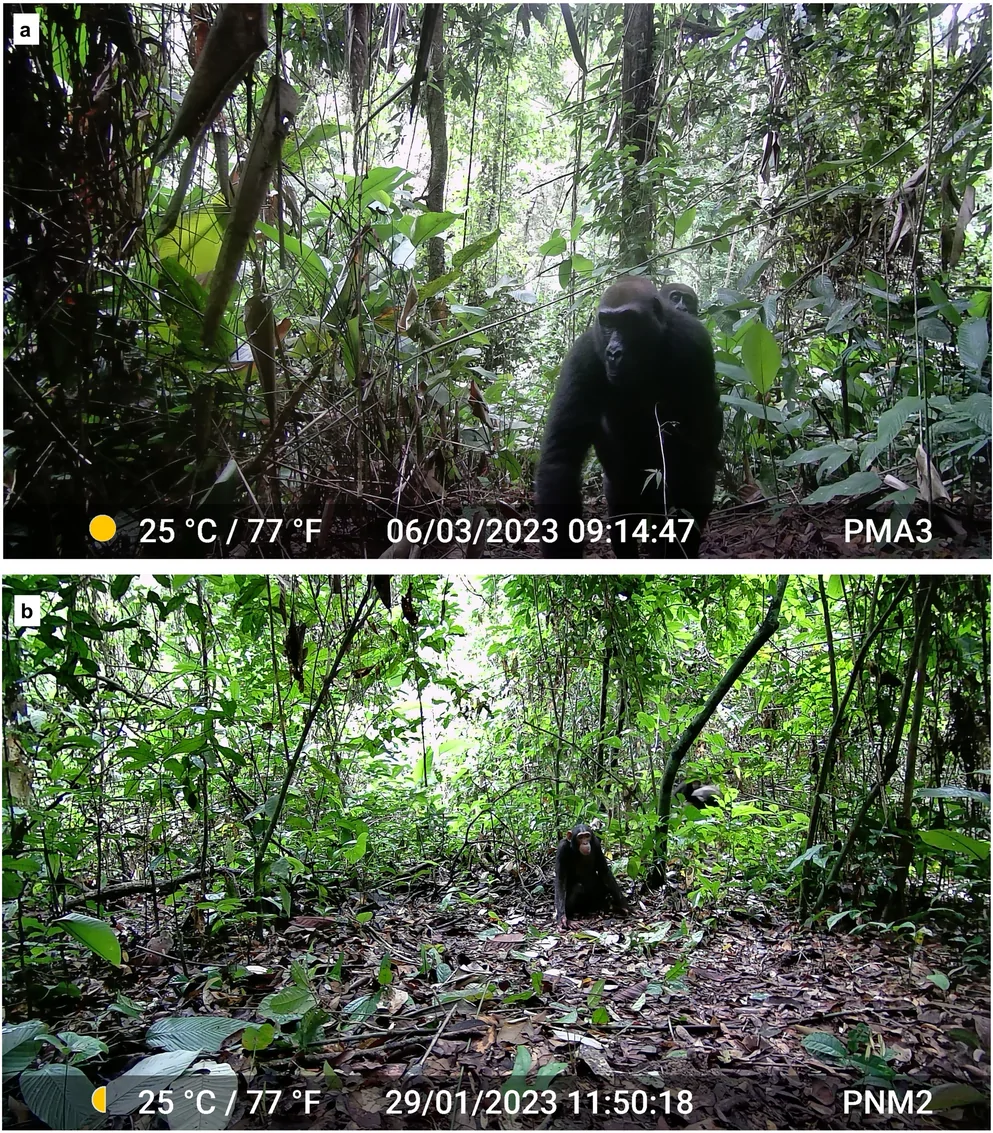
Camera trap photographs showing the first confirmed records of (**a**) a western lowland gorilla (*Gorilla gorilla gorilla*) and (**b**) a central chimpanzee (*Pan troglodytes troglodytes*) in Mayombe National Park, Angola (2023)

In 2023, data from 8 of the 17 operational camera traps confirmed the presence of great apes in MNP (Fig. 2). Gorillas were recorded in five cameras, mostly located in closer forest areas, whereas chimpanzees were detected in six cameras that were more widely dispersed across the sampling grid. Both species were recorded in three of the eight cameras with great ape detections, although never simultaneously.

**Fig. 3:**
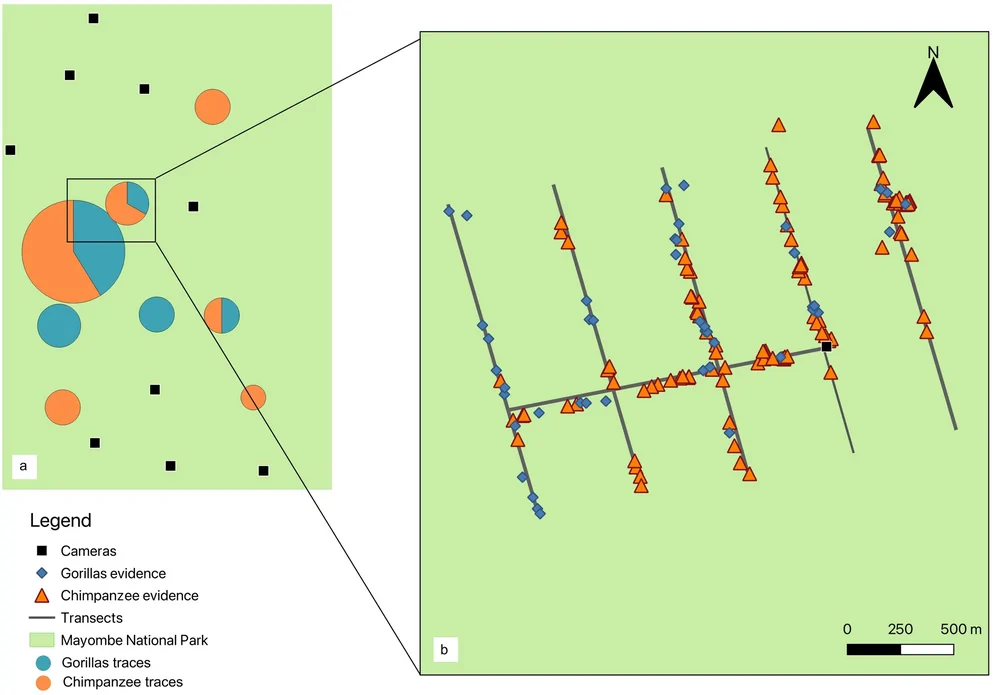
Spatial distribution of great ape detections in Mayombe National Park. **a** Locations of independent camera trap events of gorillas (blue) and chimpanzees (orange), showing areas of overlap (blue and orange) during 2023. **b** Distribution of direct and indirect evidence recorded along transects for gorillas (blue squares) and chimpanzees (orange triangles) and during 2024 transect survey. This map is intentionally generalized to avoid disclosure of sensitive locations and survey boundaries

A total of 14 independent gorilla events and 18 chimpanzee events were documented. Of these, 7 gorilla events and 10 chimpanzee events occurred at the same camera location, while the remaining detections were distributed across the other camera locations (Fig. 3a).

During the 2024 fieldwork, *ad libitum* observations confirmed the presence of both species through indirect evidence such as nests, trails, and feeding traces, particularly concentrated within the area identified by the 2023 camera trap records. Based on these findings, a core study area was defined for systematic data collection.

Within this area, six line transects provided additional confirmation of great ape presence and distribution. Across all transects, a total of 286 direct and indirect signs were recorded, including the first direct visual encounters of chimpanzees within the park (*n* = 6). Chimpanzee signs (*n* = 206) were observed more times than those of gorillas (*n* = 80) (Fig. 3b). The most common indicators of presence were feeding traces (*n* = 36) and trails (*n* = 26) for gorillas, and nests (*n* = 75) and feeding traces (*n* = 41) for chimpanzees (Table 1).

**Table 1:** Number of direct and indirect signs recorded for central chimpanzees (*Pan troglodytes troglodytes*) and western lowland gorillas (*Gorilla gorilla gorilla*) in Mayombe National Park, Angola (August-October 2024)

Type of Evidence	Central Chimpanzee (Pan troglodytes troglodytes)	Western Lowland Gorilla (Gorilla gorilla gorilla)
Feeding traces	41	36
Trails	39	26
Footprints	16	13
Nests	75	4
Faeces	7	1
Vocalizations*	24	0
Visual encounters*	6	0
Total signs	**206 (~ 73%)**	**80 (~ 28%)**

Percentages indicate the relative contribution of each species signs to the total number of records documented across all transects

*Direct evidence

## Discussion

Chimpanzee and gorilla presence has now been systematically confirmed in MNP, representing the first data-supported records of great apes in Angola. Our results show a wider distribution of chimpanzees than gorillas across the study area. Chimpanzees display greater ecology and dietary flexibility, ranging widely in search of fruit, and using tools to exploit additional resources (Sanz et al. 2022). Gorillas, by contrast, rely more on fibrous vegetation and tend to concentrate in smaller areas where herbaceous foods are abundant (Tutin and Fernandez 1993; Rogers et al. 2004; Cipolletta et al. 2007). This difference in foraging ecology may explain why chimpanzees in MNP were detected more frequently than gorillas. Alternatively, these patterns may also reflect sampling bias, as our transect coverage may have intersected a larger portion of a chimpanzee community’s home range compared to that of a smaller or more spatially cohesive gorilla group.

The documentation of both species at overlapping sites but never simultaneously suggests a pattern of spatial overlap with temporal partitioning, a coexistence dynamic also reported from other sites in Central Africa. In Loango National Park, Gabon, sympatric chimpanzees and gorillas showed overlapping habitat use but temporal separation in foraging activities (Head et al. 2011). Similarly, in Campo Ma’an, Cameroon, occupancy models revealed partial spatial overlap consistent with niche partitioning and reduced direct competition (Collins and Weladji 2024). Our findings align with this broader pattern, although both species share the same habitat space, they appear to minimize direct encounters, through temporal adjustment and differential foraging strategies. Nevertheless, sympatry does not necessarily imply neutral coexistence, as recent observations from Loango have suggested that interspecific interactions can become antagonistic under certain ecological or social pressures (Deschner and Pika 2021). Seasonal variation in fruit availability and vegetation phenology may influence the spatial concentration and temporal separation of ape activity. Whether such dynamics occur in MNP remains to be determined.

The biogeographic significance of these findings is considerable. This study confirms the presence of *Pan troglodytes troglodytes* and *Gorilla gorilla gorilla* in MNP, representing the southernmost confirmed limit of their range. Peripheral populations can hold unique ecological adaptations or genetic diversity that are absent in core populations (Fünfstück and Vigilant 2015), and their study is essential for understanding species resilience to changing environmental and anthropogenic pressures. The MNP has remained largely unexplored from a primatological perspective, leaving a critical gap between well-studied northern populations and the southern Congo River basin. Notably, Cabinda lies only a short distance south of the Congo River — the geographical barrier that separates chimpanzees (*Pan troglodytes*) from bonobos (*Pan paniscus*). This proximity positions the MNP population as potentially pivotal for reconstructing the evolutionary and biogeographic history of African great apes, including the divergence between *Pan troglodytes* and *Pan paniscus*. Ongoing genomic work will clarify whether the MNP apes represent a distinct southern population, or a continuous population connected to those across the Congo Basin.

From a conservation perspective, the systematic confirmation of both species in the Mayombe forest provides the basis for updating IUCN distribution maps (IUCN 2014) and supports previous proposals to integrate Angola into regional conservation strategies (Ron 2020). Both *Pan troglodytes troglodytes* and *Gorilla gorilla gorilla* remain highly threatened by hunting, logging, and agricultural expansion across the Congo Basin (Congo Basin International Conference 2025). The presence of sympatric great apes in Angola reinforces the need for coordinated transboundary management among Angola, the Republic of Congo, and the Democratic Republic of Congo (Tutin et al. 2005).

## Conclusion

This study provides the first systematic records of western lowland gorillas and central chimpanzees in Angola, confirming their presence in MNP. The data reveals their sympatric coexistence at the southernmost edge of their distribution, with chimpanzees detected more frequently and across more camera trap sites, while gorillas appeared more localized. These findings establish a baseline for future demographic, ecological, and genetic studies in Angola and emphasize the importance of MNP for understanding ape evolution and biogeography. By confirming Angola as part of the Central African great ape range, this study not only fills a major biogeographic gap but also opens a new frontier for research and conservation at the southern edge of great ape distribution in the continent.

Finally, our findings highlight the urgent need to integrate Angola into regional conservation strategies for great apes across the transboundary Mayombe forest.
