# Correction: A new southern limit for the distribution of African great apes: sympatric western lowland gorilla (*Gorilla gorilla gorilla*) and central chimpanzee (*Pan troglodytes troglodytes*) confirmed in Mayombe National Park, Angola

**DOI:** 10.1007/s10329-026-01282-z

**Published:** 2026-08-14

**Authors:** Mariana Cravo-Mota, Eduardo Kivete Lutondo, João Mavinga Mbenza, Graça Dala Catuti, Tatiana Simão, Dionísia Segunda, Pedro Vaz Pinto, Nuno Ferrand, Paulo Gama Mota, Susana Carvalho

**Affiliations:** 1 CIBIO Centro de Investigação Em Biodiversidade E Recursos Genéticos, InBIO Laboratório Associado, Universidade do Porto, Campus de Vairão, Vila do Conde, Portugal; 2 BIOPOLIS Program in Genomics, Biodiversity and Land Planning, CIBIO, Campus de Vairão, Vairão, 4485-661 Portugal; 3 Fundação Kissama, Luanda, Angola; 4 Maiombe National Park, Cabinda, Angola; 5 INBAC Instituto Nacional da Biodiversidade e Áreas de Conservação, Luanda, Portugal; 6 Universidade de Coimbra, Coimbra, Portugal; 7 Gorongosa National Park, Sofala, Mozambique; 8 Oxford University, Oxford, UK; 9 Faculdade de Ciências, Universidade do Porto, Porto, Portugal

**Correction: Primates** **https://doi.org/10.1007/s10329-026-01252-5**

In this article, the following errors were identified and corrected:

- The affiliations “BIOPOLIS Program in Genomics, Biodiversity and Land Planning, CIBIO, Campus de Vairão, Vairão 4485-661, Portugal” and “Fundação Kissama, Luanda, Angola” for Pedro Vaz Pinto were missing.
- The third sentence in the Abstract should read: “However, until now, no published evidence-based records have confirmed the continued presence of both species.”
- The keyword “Sympatric species” should be changed to roman (non-italic) font.
- The sentence beginning “Historical records, NGO reports and…” should read: “Historical records, NGO reports, and IUCN distribution maps have all pointed toward their occurrence, but no empirical evidence-based study had verified it until now (Ferreira et al. 1945; Ron 2005; Ron 2010; Ron 2020; IUCN 2014).”
- The species names (Gorilla gorilla gorilla and Pan troglodytes troglodytes) in the Fig. 2 caption should appear in italics.
- The sentence beginning “Chimpanzee and gorilla presence…” should read: “Chimpanzee and gorilla presence has now been systematically confirmed in MNP, representing the first data-supported records of great apes in Angola.”
- The sentence beginning “From a conservation perspective…” should read: “From a conservation perspective, the systematic confirmation of both species in the Mayombe Forest provides the basis for updating the IUCN distribution maps (IUCN 2014) and supports previous proposals to integrate Angola into regional conservation strategies (Ron 2020).”
- The reference “Ron T (2005)” was incorrect and should have been: “Ron T (2005). The Maiombe Forest in Cabinda: Conservation Efforts, 2000–2004. Gorilla Journal, 30, 18–21.”
- The references “Ron T, Golan T (2010) Angolan Rendezvous: Man and Nature in the Shadow of War. 30 Degrees South Publishers, Cape Town.” and “Ron T (2020) The struggle for survival in the Maiombe Forest continues. Gorilla Journal 61:22–25.” should be included after the reference “Ron T (2005)…”.
- In this article, Figs. 1, 2, and 3 appeared in low resolution and have now been corrected in the original publication.

The original article has been corrected.

